# Polygenic inheritance and its interplay with smoking history in predicting lung cancer diagnosis: a French-Canadian case-control cohort

**DOI:** 10.1016/j.ebiom.2024.105234

**Published:** 2024-07-05

**Authors:** Véronique Boumtje, Hasanga D. Manikpurage, Zhonglin Li, Nathalie Gaudreault, Victoria Saavedra Armero, Dominique K. Boudreau, Sébastien Renaut, Cyndi Henry, Christine Racine, Aida Eslami, Stéphanie Bougeard, Evelyne Vigneau, Mathieu Morissette, Benoit J. Arsenault, Catherine Labbé, Anne-Sophie Laliberté, Simon Martel, François Maltais, Christian Couture, Patrice Desmeules, Patrick Mathieu, Sébastien Thériault, Philippe Joubert, Yohan Bossé

**Affiliations:** a Institut universitaire de cardiologie et de pneumologie de Québec – Université Laval, Quebec City, Canada; b Anses (French Agency for Food, Environmental and Occupational Health and Safety), 22440, Ploufragan, France; c Oniris, INRAE, StatSC, 44300, Nantes, France; d Department of Molecular Medicine, Université Laval, Quebec City, Canada

**Keywords:** Lung cancer, Polygenic risk score, Genetic risk, Smoking, GWAS

## Abstract

**Background:**

The most near-term clinical application of genome-wide association studies in lung cancer is a polygenic risk score (PRS).

**Methods:**

A case-control dataset was generated consisting of 4002 lung cancer cases from the LORD project and 20,010 ethnically matched controls from CARTaGENE. A genome-wide PRS including >1.1 million genetic variants was derived and validated in UK Biobank (n = 5419 lung cancer cases). The predictive ability and diagnostic discrimination performance of the PRS was tested in LORD/CARTaGENE and benchmarked against previous PRSs from the literature. Stratified analyses were performed by smoking status and genetic risk groups defined as low (<20th percentile), intermediate (20–80th percentile) and high (>80th percentile) PRS.

**Findings:**

The phenotypic variance explained and the effect size of the genome-wide PRS numerically outperformed previous PRSs. Individuals with high genetic risk had a 2-fold odds of lung cancer compared to low genetic risk. The PRS was an independent predictor of lung cancer beyond conventional clinical risk factors, but its diagnostic discrimination performance was incremental in an integrated risk model. Smoking increased the odds of lung cancer by 7.7-fold in low genetic risk and by 11.3-fold in high genetic risk. Smoking with high genetic risk was associated with a 17-fold increase in the odds of lung cancer compared to individuals who never smoked and with low genetic risk.

**Interpretation:**

Individuals at low genetic risk are not protected against the smoking-related risk of lung cancer. The joint multiplicative effect of PRS and smoking increases the odds of lung cancer by nearly 20-fold.

**Funding:**

This work was supported by the 10.13039/100015751CQDM and the IUCPQ Foundation owing to a generous donation from Mr. Normand Lord.

**Research in context**

**Evidence before this study**

We searched PubMed up to November 30, 2023 for articles published in English on polygenic risk score studies in the field of lung cancer using the search terms (“polygenic risk score” OR “polygenic score” OR “PRS”) AND “lung cancer”. Relevant studies were also identified in review articles and by examining the references section of identified articles. Previous PRSs developed in the field were derived from a limited set of genome-wide significant and subthreshold variants. These PRSs have achieved significant prediction of prevalent and incident lung cancer cases beyond well-established clinical risk factors. In the context of lung cancer screening programs, PRSs have demonstrated their potential value in informing the optimal timing for screening, but this will require further confirmation. Mature lung cancer PRSs ready for clinical applications are not available yet.

**Added value of this study**

We built a new case-control cohort of patients with lung cancer, independent from genome-wide association studies that reported lung cancer variants, to externally benchmark the performance of previously published PRSs. We also generated a genome-wide PRS in lung cancer with more than 1.1 million variants and demonstrated that it numerically outperformed previous PRSs. The genome-wide PRS is a significant predictor of lung cancer associated with a 2-fold odds of lung cancer among individuals in the top vs. bottom quintile of the PRS distribution. Smoking is associated with approximately a 10-fold increased risk of lung cancer regardless of the polygenic background. The PRS and smoking-related risk of lung cancer multiply to roughly 20-fold in high genetic risk (top quintile) individuals who smoked compared to low genetic risk (bottom quintile) individuals with no smoking history.

**Implications of all the available evidence**

This study contextualizes the current predictive ability and disease discrimination performance of a genome-wide PRS in lung cancer. Although statistically significant and independent from conventional lung cancer risk factors, the effect of the PRS is incremental as part of an integrated lung cancer risk model. No subset of individuals is genetically protected against the smoking-related risk of lung cancer. In relation with smoking history, the resulting impact of the lung cancer risk doubling effect of the high vs. low genetic risk groups is smoking-dependent with cumulative risk passing from 1-fold to 2-fold in never-smokers and 10-fold to 20-fold in ever-smokers. The extent of risk stratification benefits associated with the PRS is thus more meaningful in ever smokers, which may inform PRS implementation efforts.

## Introduction

A genetic component to lung cancer has long been recognized.^1^, ^2^, ^3^ We have completed the largest genome-wide association study (GWAS) on lung cancer in individuals of European ancestry as part of the International Lung Cancer Consortium (ILCCO) including 29,266 lung cancer cases and 56,450 controls.^4^ This GWAS highlighted the genetic heterogeneity across histological subtypes of lung cancer and reported novel loci for lung cancer per se, adenocarcinoma and squamous cell carcinoma. Combined with other published GWAS on lung cancer, a total of 45 susceptibility loci have been identified so far.^5^ Individually, the lung cancer-associated loci have small effect sizes and thus limited clinical utility. Collectively, however, they can potentially be grouped into a polygenic risk score (PRS) to delineate a subgroup of individuals at higher genetic risk of lung cancer. The clinical utility of PRS in lung cancer risk assessment is emerging,^6^ but requires further investigations.

Dai et al.^7^ demonstrated that a PRS constructed from statistically significant GWAS variants could provide risk prediction that is independent from conventional predictors such as smoking status. In their large prospective study, namely the China Kadoorie Biobank, the hazard ratio predicting the incidence of lung cancer was 2.37 in individuals in the top 5% vs. the bottom 5% of the PRS distribution. In the context of improving the efficiency of lung cancer screening, Hung et al.^8^ showed that an individual's genetic background captured by PRS may inform the optimal timing for screening. More explicitly, the individual's age for reaching the threshold to be recommended for screening, which was set in that study at 1.5% lung cancer absolute risk within the next 5 years, can vary by 4–8 years depending on the genetic makeup. Other lung cancer PRSs have been reported using a variety of methods for selecting and weighting variants.^9^, ^10^, ^11^, ^12^, ^13^, ^14^, ^15^, ^16^, ^17^ However, none have used the full spectrum of genetic risk, i.e. genome-wide data, to derive the PRS. In addition, in the context of lung cancer, where tobacco smoking is the main risk factor, it remains unclear how the PRS could amplify or mitigate the risk associated with smoking. Greater effects of the PRS were observed in individuals with a smoking history in some studies^15^^,^^18^ and in individuals who never smoked in others.^19^^,^^20^

In this study, we hypothesized that our knowledge of the genetic factors underlying lung cancer has reached a point where we can develop a PRS to improve risk prediction and delineate individuals at high genetic risk of lung cancer for earlier detection and prevention. The goals of this study were three-fold: first, to develop and validate distinct PRSs derived from GWAS-identified variants or a genome-wide method and benchmark their performance in a new case-control cohort independent from previous lung cancer GWAS; second, to compare the lung cancer risk prediction ability and diagnostic discrimination performance of the PRS beyond non-genetic factors and construct an integrated risk score combining both clinical and genetic data; and third, to evaluate whether the contribution of the PRS in lung cancer risk assessment is influenced by smoking status.

## Methods

### Study design

An overview of the study workflow and key results are presented in Supplementary Figure S1. This study was built on effect size estimation from the largest GWAS on lung cancer published in individuals of European descent (29,266 cases and 56,450 controls).^4^ Two independent cohorts were then used to identify the best performing PRS for lung cancer risk prediction. First, data from UK Biobank was divided into a training set (1/3 of cases and controls) and a validation set (2/3 of cases and controls). Second, testing was performed with an ethnically-matched case-control dataset (LORD/CARTaGENE). The recruitment and data collection of each dataset is described below.

### The LORD cohort

#### Lung cancer patients

Lung Oncology Research & Discovery (LORD) is an ongoing translational research program towards precision medicine in lung cancer diagnosis, management and treatment conducted at the Quebec Heart and Lung Institute (IUCPQ-UL or “Institut universitaire de cardiologie et de pneumologie de Québec—Université Laval”), located in Quebec City, Canada. LORD consists of a single site case-only cohort based on a 20-year data collection (1999–2021) of patients who underwent lung cancer surgery at IUCPQ-UL. Lung cancer diagnosis was confirmed by pathological examination using resected specimens. Clinical data including demographics, pathology report, surgical procedure and self-reported smoking history at the time of surgery were collected in a local database.

#### Genotyping and quality control

Blood samples were collected at surgery and DNA was extracted from frozen buffy coat. For the majority of cases (n = 3815), genotyping was performed using the Illumina Global Screening Array (GSA) version 3 BeadChip with the multi-disease (MD) drop-in panel. A smaller subset of patients (n = 268) were genotyped as part of a prior project using the Illumina GSAv1 + MD BeadChip. Quality controls (QC) were performed excluding low-quality genetic variants with 10th percentile of Illumina GenCall score ≤0.1, call rate <0.97%, Hardy–Weinberg equilibrium P < 1E-7, minor allele frequency (MAF) <1%, or duplicate variants sharing the same base-pair coordinate. A total of 493,588 and 495,183 variants passed QC checks on the GSAv3 and GSAv1 platforms, respectively. Low quality DNA samples were also filtered out after consideration for the genotype completion rate <95%, genotypic and phenotypic sex mismatch, unexpected duplicates and genetic relatedness (first-degree relatives) evaluated by identity-by-state using PLINK, outliers based on the inbreeding coefficients (F >10 standard deviation from the mean), and genetic background outliers detected by principal component analysis with HapMap subjects as population reference panel. A total of 3738 and 265 samples passed QC checks on the GSAv3 and GSAv1 platforms, respectively. Genotyping data from the two platforms were then combined using Rayner's script (https://www.well.ox.ac.uk/∼wrayner/strand/index.html) and the corresponding manifest files providing the strand orientation and position of variants. One duplicate sample on GSAv3 and GSAv1 was excluded and 449,454 common variants were carried forward for subsequent analyses. After the QC filters, 4002 cases of European descent confirmed at genotyping were available for subsequent analyses. Supplementary Table S1 provides a summary of genotyping QCs for variants and samples.

### CARTaGENE

#### Control individuals

CARTaGENE is a population-based project including residents from the province of Quebec enrolled at the age of 40–69^21^ (www.cartagene.qc.ca). Participants were not recruited based on diseases or health, but represent a random selection among the population. Individuals with health and genotyping data were considered in this study. Genotyping data were obtained for all participants of the cohort that provided a biological sample. In addition, self-reported and incident cases of lung cancer were excluded in order to obtain a control-only cohort. Smoking history in CARTaGENE was captured by a lifestyle questionnaire. Ever and never smokers were identified by a positive or negative answer to the question “In your lifetime have you smoked a total of 100 cigarettes or more?”

#### Genotyping and quality control

The initial genotyping data consisted of 29,337 individuals genotyped using five versions of the Illumina GSA BeadChip. Standard QCs as described above were performed within each subset and variants common to all GSA versions were merged using Rayner's script and the corresponding manifest files into a final set of 460,249 variants for 25,653 subjects.

### The LORD/CARTaGENE cohort

Combining LORD and CARTaGENE has generated an ethnically-matched retrospective cohort of prevalent lung cancer cases and controls.

#### Selection of ethnically matched cases and controls

Considering the different origin of cases from LORD (hospital-based) and controls from CARTaGENE (population-based), we have matched each case to a maximum number of controls using ancestry-based principal components (PC) 1 and 2. This has resulted in the selection of five controls per case, leading to a final sample size of 4002 cases and 20,010 controls of European ancestry confirmed by genotyping (Supplementary Figure S2). Genotyping data from LORD and CARTaGENE were merged into a final set of 388,262 common variants.

#### Imputation

Lung cancer cases from LORD (n = 4002) and controls from CARTaGENE (n = 20,010) were imputed together with the Michigan Imputation Server^22^ using TOPMed data (build GRCh38) as reference set. Only common variants between LORD and CARTaGENE (n = 388,262) were used for the imputation. Following imputation, variants with an imputation quality score (INFO) <0.3, MAF <0.001, or MAF x imputation quality x n cases <10 (as advised by Roselli et al.^23^) were removed from further analysis. A total of 11,547,025 imputed variants passed QC.

#### Genetic association tests

The genetic association analysis was performed using SAIGE (Scalable and Accurate Implementation of GEneralized mixed model, version 0.45, https://github.com/weizhouUMICH/SAIGE).^24^ Analyses were adjusted for age, sex, and the first 10 ancestry-based PC. We applied the leave-one-chromosome-out (LOCO) scheme (LOCO = TRUE). The genomic inflation factor for the case-control analysis was 0.989. The quantile–quantile plot is showed in Supplementary Figure S3. The genome-wide significant P value cut-off was set to 5E-8.

### UK Biobank

UK Biobank is an open access resource of nearly 500,000 participants enrolled at the age of 40–69 and prospectively evaluated for a range of health-related outcomes.^25^ The definition of lung cancer in this study relies on International Classification of Diseases (ICD) codes, ICD9 and ICD10, and self-reported related codes described previously^26^ (Supplementary Table S2). Genotyping data are derived from the Affymetrix UK BiLEVE or the UK Biobank Axiom Arrays. Phasing and imputation were performed centrally using the Haplotype Reference Consortium and merged UK10K and 1000 Genomes phase 3 reference panels. Samples with call rate <95%, outlier heterozygosity rate, sex mismatch, non-White British ancestry, samples with excess third-degree relatives (>10), or not used for relatedness calculation were excluded. Variants with an imputation quality score (INFO) ≤0.3 or MAF <0.0001 were removed. Using the aforementioned definition of lung cancer and quality control filters, 5419 lung cancer cases and 403,003 controls of White British ancestry were identified.

### Genome-wide PRS

We developed different PRSs to evaluate their association with lung cancer. The variant-level effect size estimation was obtained from summary statistics of a GWAS on lung cancer including 29,266 cases and 56,450 controls of European descent.^4^ In this GWAS, the genetic association model was adjusted for three ancestry-based PC and assumed an additive mode of inheritance. The LDpred2 computational algorithm^27^ was used to derive the PRS. White British individuals from UK Biobank (n = 408,422) was used as the reference panel to model linkage disequilibrium. Only variants included in the Phase 3 HapMap Consortium (∼1.2 million) were kept for the PRS derivation as recommended.^27^ The UK Biobank cohort (n = 5419 lung cancer cases and 403,003 controls) was then divided in 1/3 and 2/3 to train and validate our model, respectively, and identify the best set of genetic variants. Different models were evaluated in LDpred2 including automatic, infinitesimal, and grid with or without sparse. The model showing the strongest association with lung cancer was selected using logistic regression adjusted for age, sex, and ancestry-based PC1-10. The PRS was then calculated, standardized and tested in the LORD/CARTaGENE cohort. Supplementary Figure S4 illustrates the workflow to develop the genome-wide PRS.

### GWAS-SNP PRS

PRSs derived from two sources of previous GWAS-nominated loci were also constructed using the 45 lung cancer susceptibility loci tabulated by Bossé and Amos^5^ and the latest GWAS on lung cancer by Byun et al.^28^ For each source, two versions of the PRS were generated: 1) unweighted, representing the sum of risk alleles, and 2) weighted, representing the sum of risk alleles weighted based on their effect sizes [log (OR)]. For the 45 lung cancer susceptibility loci, the risk allele and effect size were derived from previous lung cancer GWAS as indicated in Supplementary Table S3. For loci reported by more than one publication (e.g., 15q25), the median effect size (derived from the reported effect size of all GWA studies) was selected. For one of the 45 loci, the risk allele and effect size were not available leading to a 44-variant PRS labelled Bossé_44_unweighted and Bossé_44_weighted. For the PRS derived by Byun et al.,^28^ non-overlapping variants claimed statistically significant in the discovery study across histological subtypes (overall lung cancer, adenocarcinoma, squamous cell carcinoma and small cell lung cancer) were selected (Supplementary Table S4), which led to a 38-variant PRS labelled Byun_38_unweighted and Byun_38_weighted. The effect sizes were taken from the European association results in order to match the ancestry of the LORD/CARTaGENE cohort. PRSs were calculated using the riskScore function in the R package PredictABEL.^29^

### Benchmarking with previously published lung cancer PRSs

The genome-wide PRS and PRSs from GWAS-identified loci were compared to previously reported PRSs for lung cancer. A total of ten PRSs were evaluated. From these ten PRSs, nine were recently summarized by Lebrett et al.^17^ (including the list of variants and corresponding weights) and one by Zhu et al.^18^ Variants and risk alleles were matched to the LORD/CARTaGENE cohort and proxy variants (r^2^ >0.5) were identified as needed using the LDproxy tool.^30^ The weighted [log (OR)] sum of allele counts for each PRS was obtained using the riskScore function in the R package PredictABEL.^29^ The final set of PRSs and variants available in LORD/CARTaGENE includes the 19-variant by Dai et al.^7^ (henceforth referred as Dai_19), 6-variant by Shi et al.^14^ (Shi_6), 102-variant by Graff et al.^12^ (Graff_102), 19-variant by Jia et al.^16^ (Jia_19), 14-variant and 19-variant by Fritsche et al.^13^ (Fritsche_14 and Fritsche_19), 35-variant and 125-variant by Hung et al.^8^ (Hung_35 and Hung_125), 32-variant by Zhang et al.^15^ (Zhang_32) and 22-variant by Zhu et al. (Zhu_22).^18^ The final set of variants and weights for the ten PRSs are listed in Supplementary Table S5.

### Statistics

Clinical characteristics of participants were summarized by lung cancer status as mean ± standard deviation for continuous variables and as percentage for discrete variables. Logistic regression and receiver operating characteristic (ROC) curves were used to evaluate the performance of the PRS to discriminate between individuals with and without lung cancer. Multivariable logistic regressions were evaluated to determine whether the PRS is an independent predictor of lung cancer beyond clinical data. The predictors of lung cancer risk were combined into an integrated risk model combining both clinical and genetic data. Variables included in the model were PRS, age, sex, smoking status (ever vs. never), body mass index (BMI) and the first ten ancestry-based PC. The models with and without the PRS were compared using the likelihood ratio test and the continuous (category-free) net reclassification index (NRI). Genetic risk groups were defined as low (<20th percentile), intermediate (20–80th percentile) and high (>80th percentile) PRS. Odds ratios for lung cancer were calculated per standard deviation of PRS, across PRS quintiles (cumulative PRS distribution split into five equally-sized subsets), genetic risk groups and stratified by smoking status. Odds ratios were also calculated across combination of genetic risk and smoking subgroups with the low genetic risk and no smoking as the reference. Interaction analysis was performed by logistic regression with the PRS as a continuous variable, smoking status and multiplicative interaction (PRS∗smoking) terms. Statistical analyses were carried out using the R statistical software version 4.3.2.

### Heritability

LD-score regression was used to estimate SNP-heritability for lung cancer.^31^ To obtain heritability on the liability scale, we provided sample and population prevalence of 16.7% (--samp-prev 0.167) and 4.1% (--pop-prev 0.041) (Canadian Cancer Statistics 2022, cancer.ca/Canadian-Cancer-Statistics-2022-EN), respectively. Only variants with INFO >0.9 in LORD/CARTaGENE (n = 8,897,496) were included in the SNP-heritability analysis.

### Ethics

All patients from the LORD cohort provided written informed consent to participate in our local biobank and the study was approved by the ethics committee of the Institut universitaire de cardiologie et de pneumologie de Québec – Université Laval (#21871). Data from CARTaGENE and UK Biobank are publicly available through access requests. Ethical approval and individual consent were obtained from the original studies by their corresponding ethical review committees. Access to CARTaGENE in this project has been approved under data application number 890519. Access to UK Biobank has been approved under data application number 25205.

### Role of funders

The funding sources had no role in study design, data collection, data analysis, data interpretation, or writing of the report. YB was responsible for the decision to submit the manuscript.

## Results

### Lung cancer GWAS in LORD/CARTaGENE

Demographics and clinical characteristics stratified by cases and controls are presented in Table 1, and further stratified by sex are showed in Supplementary Table S6. Cases were from the LORD project and consist of 4002 patients who underwent lung cancer surgery and passed genotyping quality control filters. The mean age at surgery was 65 years and 52% of patients were females. A majority of patients had early-stage disease including 2399 (60%) with pathological stage I. The most common histologic subtypes were adenocarcinoma (63%) and squamous cell carcinoma (23%). Most patients were ever smokers (93%). Controls were from a population-based cohort of individuals living in the province of Quebec and were ethnically-matched in a 5:1 ratio to the lung cancer cases from LORD. Sex and body mass index were relatively well balanced between cases and controls. However, controls were younger (average of 55 years) and had a greater proportion of individuals who never smoked (7% in cases vs. 41% in controls).

**Table 1:** Demographics and clinical characteristics of lung cancer cases (LORD) and controls (CARTaGENE).

	LORD	CARTaGENE
	n = 4002	n = 20,010
Sex		
Male	1924 (48.1%)	9056 (45.3%)
Female	2078 (51.9%)	10,954 (54.7%)
Age (years)	64.7 ± 8.78	54.6 ± 7.85
BMI (m^2^/kg)	26.7 ± 5.25	27.6 ± 5.90
Smoking status		
Ever	3734 (93.3%)	11,596 (58.0%)
Never	268 (6.7%)	8151 (40.7%)
Missing	0	263 (1.3%)
Histology		
Adenocarcinoma	2534 (63.3%)	NA
Squamous cell carcinoma	920 (23.0%)	NA
Neuroendocrine tumor	277 (6.9%)	NA
Others	271 (6.8%)	NA
Pathological stages		
I	2399 (60%)	NA
II	789 (19.7%)	NA
III	630 (15.7%)	NA
IV	43 (1.1%)	NA
Missing	141 (3.5%)	NA

Continuous variables are presented as mean ± SD. Discrete variables are presented as n (%).

BMI, body mass index.

**Fig. 1:**
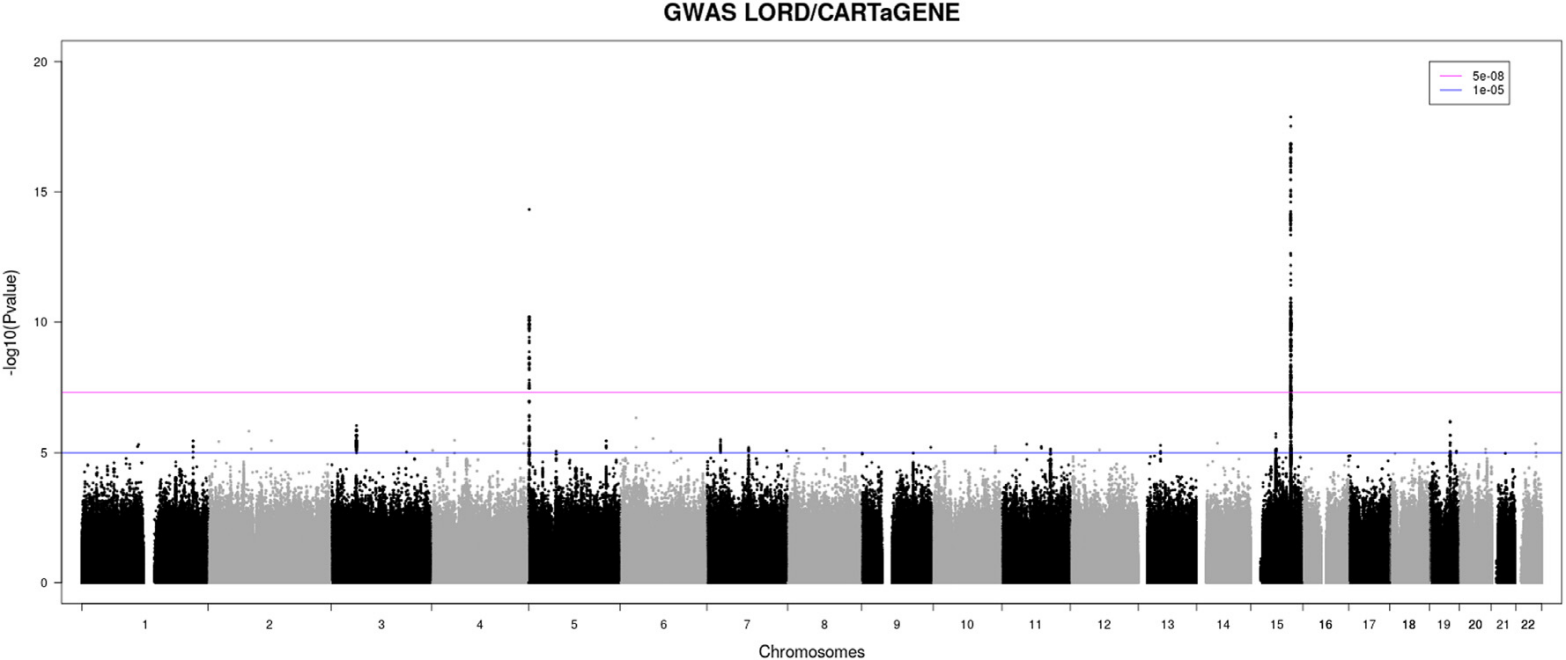
Manhattan plot of the GWAS LORD/CARTaGENE including 4002 lung cancer cases and 20,010 controls. The y axis represents P values in -log10 scale [mixed model-based association test]. The horizontal blue and magenta lines indicate P values of 1E-5 and 5E-8, respectively.

The GWAS analysis of the LORD/CARTaGENE cohort includes 4002 cases and 20,010 ethnically-matched controls. A total of 11,547,025 genetic variants were available for genetic association testing following standard quality controls and imputation. We observed no evidence of inflation in the test statistics with λ = 0.989. The SNP-heritability on the liability scale was estimated at 18.5%, assuming a disease prevalence of 4.1%. In total, 338 SNPs reached genome-wide significance (P_GWAS_ <5.0E-8) at two well-known lung cancer loci, 15q25 and 5p15 (Fig. 1). The sentinel variants at both loci showed directional consistency in effect size compared to previous lung cancer GWAS of European descent^4^ (15q25: rs12914385/chr15:78606381:C:T, risk allele = T, OR = 1.28, P = 1.33E-18 & 5p15: rs2853677/chr5:1287079:G:A, risk allele = G, OR = 1.25, P = 4.75E-15 [mixed model-based association test]). Although no new lung cancer loci were found in this French-Canadian population, the results on 15q25 and 5p15 serve as positive controls for the GWAS analysis. LORD/CARTaGENE was not part of the previous GWAS on lung cancer and thus represents an independent dataset to evaluate the clinical value of PRS and externally benchmark the performance of previously reported lung cancer PRSs.

### Polygenic susceptibility to lung cancer

**Fig. 2:**
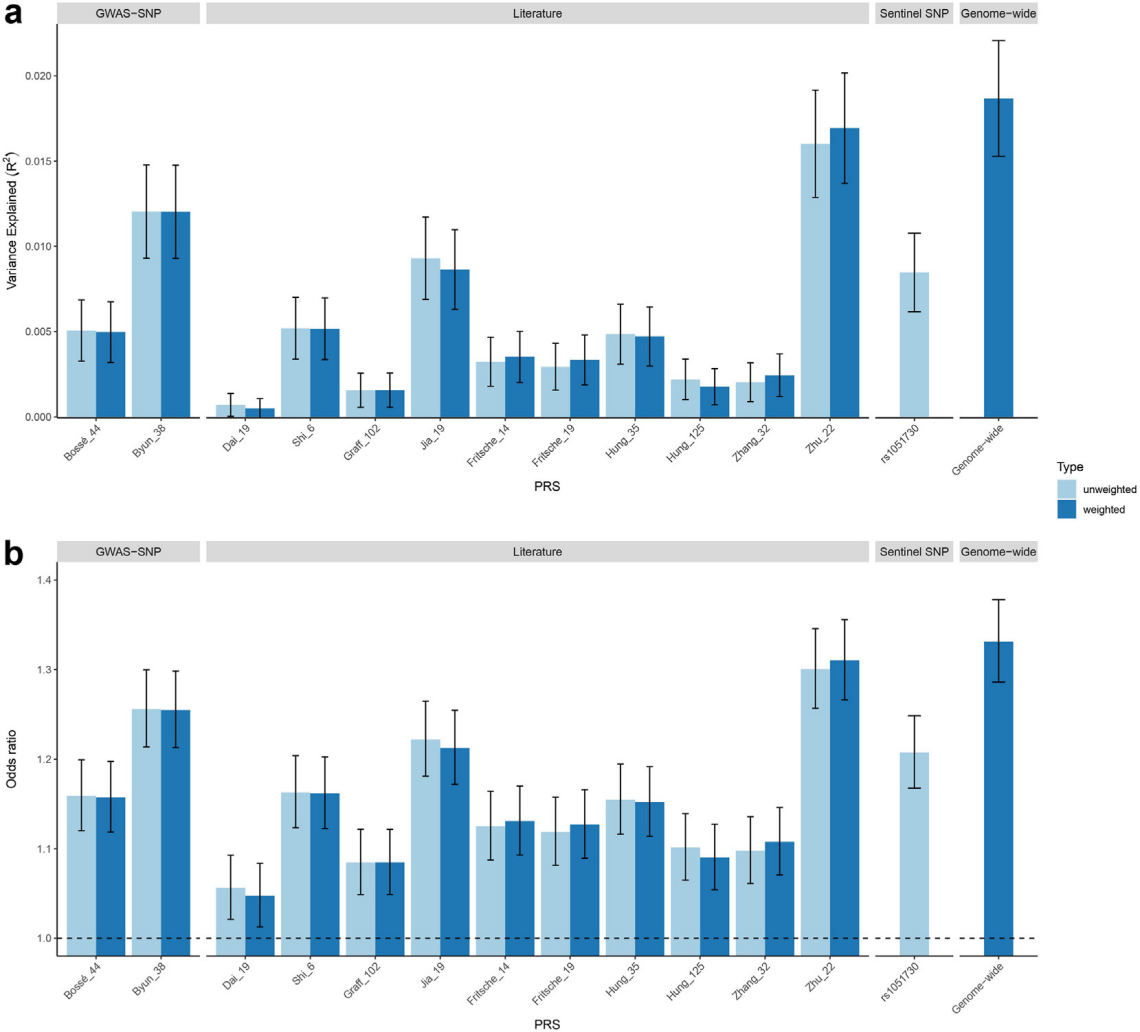
Benchmarking the various PRSs in univariable logistic regression. a) Variance explained by the PRSs along with 95% confidence intervals. b) Odds ratios of lung cancer per 1 standard deviation increase in PRSs along with 95% confidence intervals. The various PRSs includes: Two PRSs developed in this study from GWAS-nominated loci obtained from Bossé & Amos^5^ and Byun et al.,^28^ named Bossé_44 and Byun_38; Ten PRSs obtained from the literature, named Dai_19,^7^ Shi_6,^14^ Graff_102,^12^ Jia_19,^16^ Fritsche_14 and Fritsche_19,^13^ Hung_35 and Hung_125,^8^ Zhang_32,^15^ and Zhu_22^18^; A PRS derived from a single sentinel variant (rs1051730) on 15q25; A genome-wide PRS generated in this study. Horizontal dashed line set at OR of 1 in panel b.

The genome-wide PRS including 1,143,555 variants was trained in a first set (1/3 of the cohort) and validated in a second independent set (2/3) of UK Biobank. Results of the validation step are provided in Supplementary Figure S5. The genome-wide PRS was then calculated and tested for association with lung cancer in LORD/CARTaGENE. To benchmark its performance, we compared the phenotypic variance explained (R^2^) to previously published PRSs and to the 44-variant (Bossé_44) and 38-variant (Byun_38) PRSs from GWAS-identified loci. The genome-wide PRS explained 1.9% of the phenotypic variation (Fig. 2a). In comparison, the Bossé_44 and Byun_38 explained 0.5% and 1.2% of phenotypic variance, respectively. Note that among variants included in Bossé_44 and Byun_38, nine (20%) and 13 (34%) reached a P value < 0.05 [mixed model-based association test] for association with lung cancer in LORD/CARTaGENE (Supplementary Tables S3 and S4). The previous PRSs explaining the highest proportion of variance were Zhu_22_weighted (1.7%) and Jia_19_unweighted (0.9%) (Fig. 2a). The predicting ability of the various PRSs was also assessed by the risk scores effect size, i.e. OR per 1 standard deviation increase in PRS (Fig. 2b). The highest numerical OR was derived from the genome-wide PRS (OR = 1.33, 95% CI = 1.29–1.38), followed by Zhu_22_weighted (OR = 1.31, 95% CI = 1.27–1.35) and Jia_19_unweighted (OR = 1.22, 95% CI = 1.18–1.27). The 44-variant PRS performed relatively poorly in this study (OR = 1.16, 95% CI = 1.12–1.20), whereas the 38-variant PRS performance (OR = 1.26, 95% CI = 1.21–1.30) ranged between the genome-wide PRS and Jia_19. Stratified by age (< or ≥ 65 years old), the effect size of the genome-wide PRS was numerically higher in younger (1.38, 95% CI = 1.31–1.44, n = 1806) compared to older (1.30, 95% CI = 1.24–1.36, n = 2196) patients relative to the same control group (n = 20,010). Finally, we tested disease discrimination using ROC curves for all PRSs (Supplementary Table S7) and the highest AUC was observed for the genome-wide PRS (see below). The subsequent analyses were thus restricted to this PRS.

**Fig. 3:**
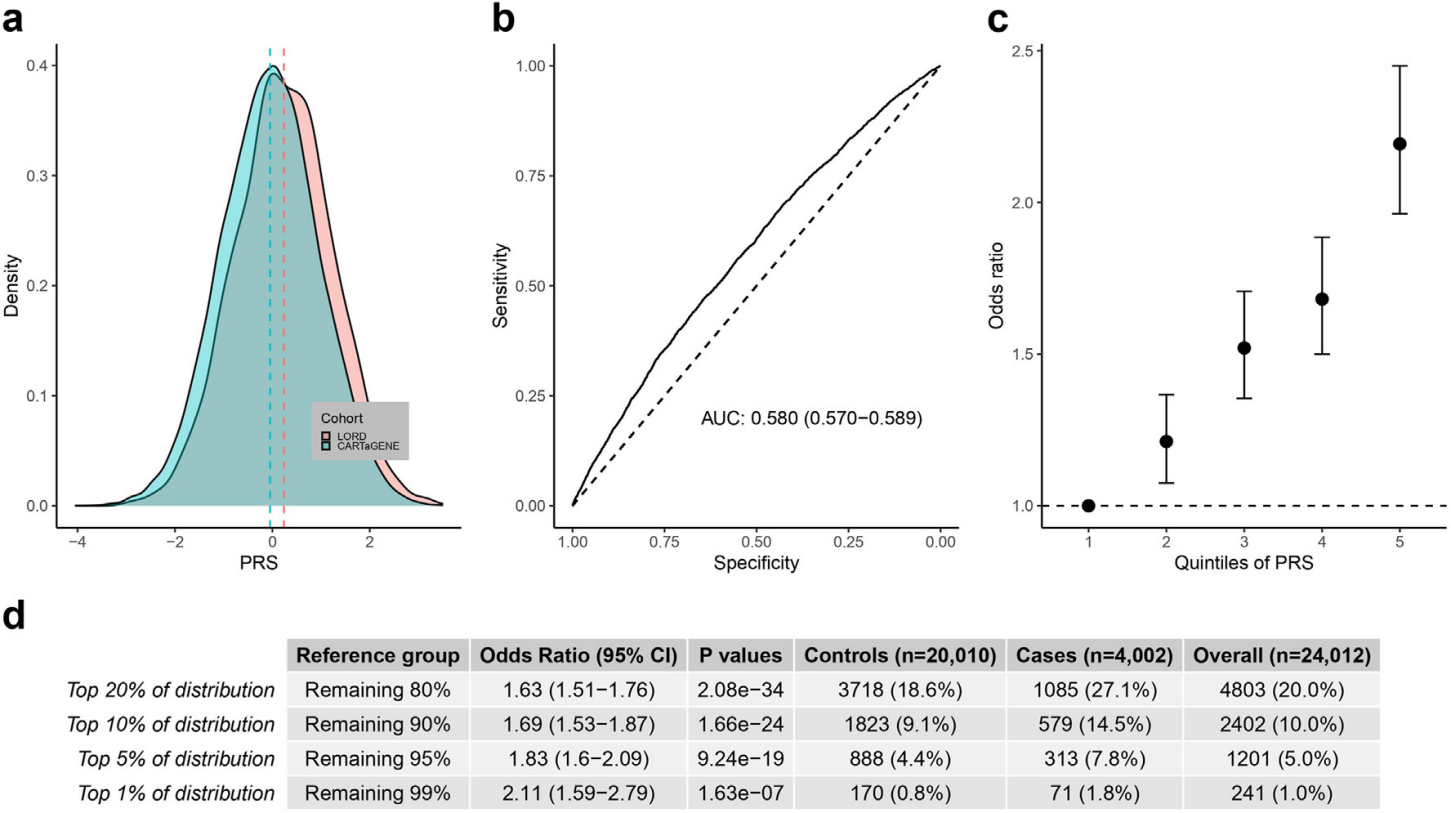
Distribution and association of the genome-wide PRS with lung cancer in LORD/CARTaGENE. a) Distribution of PRS among cases and controls. b) Receiving operating characteristic curve showing the value of the PRS at discriminating between lung cancer cases and controls. c) Odds ratio of lung cancer per quintile increase in the PRS along with 95% confidence intervals. Quintile 1 as the reference including 20% of individuals with the lowest PRS. d) Risk of lung cancer according to different genome-wide PRS thresholds. All panels present univariable analysis.

The genome-wide PRS distribution differs between cases and controls (Fig. 3a). More cases (27%) were in the top quintile of the PRS distribution compared to controls (19%), whereas more controls (21%) than cases (14%) were in the bottom quintile (Table 2). This PRS alone (without covariates) is able to discriminate lung cancer cases from controls with an AUC of 0.580 (95% CI = 0.570–0.589) (Fig. 3b). The OR for lung cancer in individuals in the highest PRS quintile compared to the lowest PRS quintile was 2.19 (P = 7.76E-44 [logistic regression]) (Fig. 3c). The proportions of cases and controls that exceed more extreme tails of the PRS distribution were then compared. The odds ratios of lung cancer at higher PRS thresholds representing the >90th, 95th and 99th percentiles were respectively evaluated at 1.69, 1.83, and 2.11 (Fig. 3d). Results presented in Fig. 3 stratified by sex are provided in Supplementary Figures S6 and S7. The results for females and males were relatively consistent, but the progressive increase in odds ratios with higher PRS thresholds was more pronounced in males.

**Table 2:** The number of cases (LORD) and controls (CARTaGENE) according to the genetic risk groups.

	Low PRS <20th	Intermediate PRS 20–80th	High PRS >80th	P value^a^
LORD	564 (14.1%)	2353 (58.8%)	1085 (27.1%)	1.57E-45
CARTaGENE	4239 (21.2%)	12,053 (60.2%)	3718 (18.6%)	

a Chi-squared test.

The value of the PRS was then gauged within an integrated risk model including well-established risk factors for lung cancer. Adding smoking as part of the risk model substantially improved the AUC to 0.718 (95% CI = 0.710–0.725), but without affecting the effect size and statistical significance of the PRS (Table 3). In a full integrated risk model including age, sex, BMI, smoking and the first 10 ancestry-based PC, the OR associated with the PRS was still 1.32 and statistically significant (AUC for the full model = 0.893) (Table 3). This suggests that the PRS is associated with lung cancer independently of conventional risk factors. The integrated risk model was then compared with and without the PRS. Adding the PRS to the full clinical model slightly increased the AUC from 0.890 (95% CI = 0.885–0.896) to 0.893 (95% CI = 0.887–0.898), indicating that the effect of the PRS is statistically significant (P < 2.2e-16 [likelihood ratio test]), but incremental within an integrated risk model combining both clinical and genetic data. In addition, integrating the PRS into the full clinical model significantly improved the continuous net reclassification index (NRI = 0.22, 95% CI = 0.18–0.27) with 10.0% of lung cancer cases correctly assigned a higher predicted risk and 12.2% of controls correctly assigned a lower predicted risk. The multiplicative interaction term between PRS and smoking into the full model with PRS was not statistically significant (P = 0.11 [logistic regression]) and did not improve the model performance (R^2^ = 0.49, AUC = 0.893 95% CI = 0.888–0.899).

**Table 3:** Association of PRS with lung cancer in LORD/CARTaGENE adjusted or not for covariates.

	Effect (SE)	OR (95% CI)	P value	R^2^	AUC (95% CI)
Genome-wide PRS^a^	0.289 (0.018)	1.33 (1.29–1.38)	3.88E-59	0.02	0.580 (0.570–0.589)
PRS and smoking^b^					
PRS	0.280 (0.018)	1.32 (1.28–1.37)	1.13E-51	0.16	0.718 (0.710–0.725)
Ever smoker	2.276 (0.065)	9.74 (8.60–11.09)	7.66E-269		
Full model without PRS^c^					
Age	0.152 (0.003)	1.16 (1.16–1.17)	0.00	0.48	0.890 (0.885–0.896)
Sex, male	−0.283 (0.045)	0.75 (0.69–0.82)	3.38E-10		
BMI	−0.044 (0.004)	0.96 (0.95–0.97)	6.32E-24		
Ever smoker	2.214 (0.072)	9.15 (7.96–10.57)	5.89E-207		
Full model with PRS^c^					
PRS	0.280 (0.023)	1.32 (1.27–1.38)	2.72E-35	0.49	0.893 (0.887–0.898)
Age	0.152 (0.003)	1.17 (1.16–1.17)	0.00		
Sex, male	−0.274 (0.045)	0.76 (0.70–0.83)	1.80E-09		
BMI	−0.044 (0.004)	0.96 (0.95–0.97)	1.22E-23		
Ever smoker	2.211 (0.072)	9.13 (7.93–10.54)	1.46E-204		

P value calculated treating the PRS as a continuous variable.

a Univariable logistic regression.

b Multivariable logistic regression.

c Multivariable logistic regression adjusted for the first 10 ancestry-based PCA.

### The interplay between PRS and smoking

**Fig. 4:**
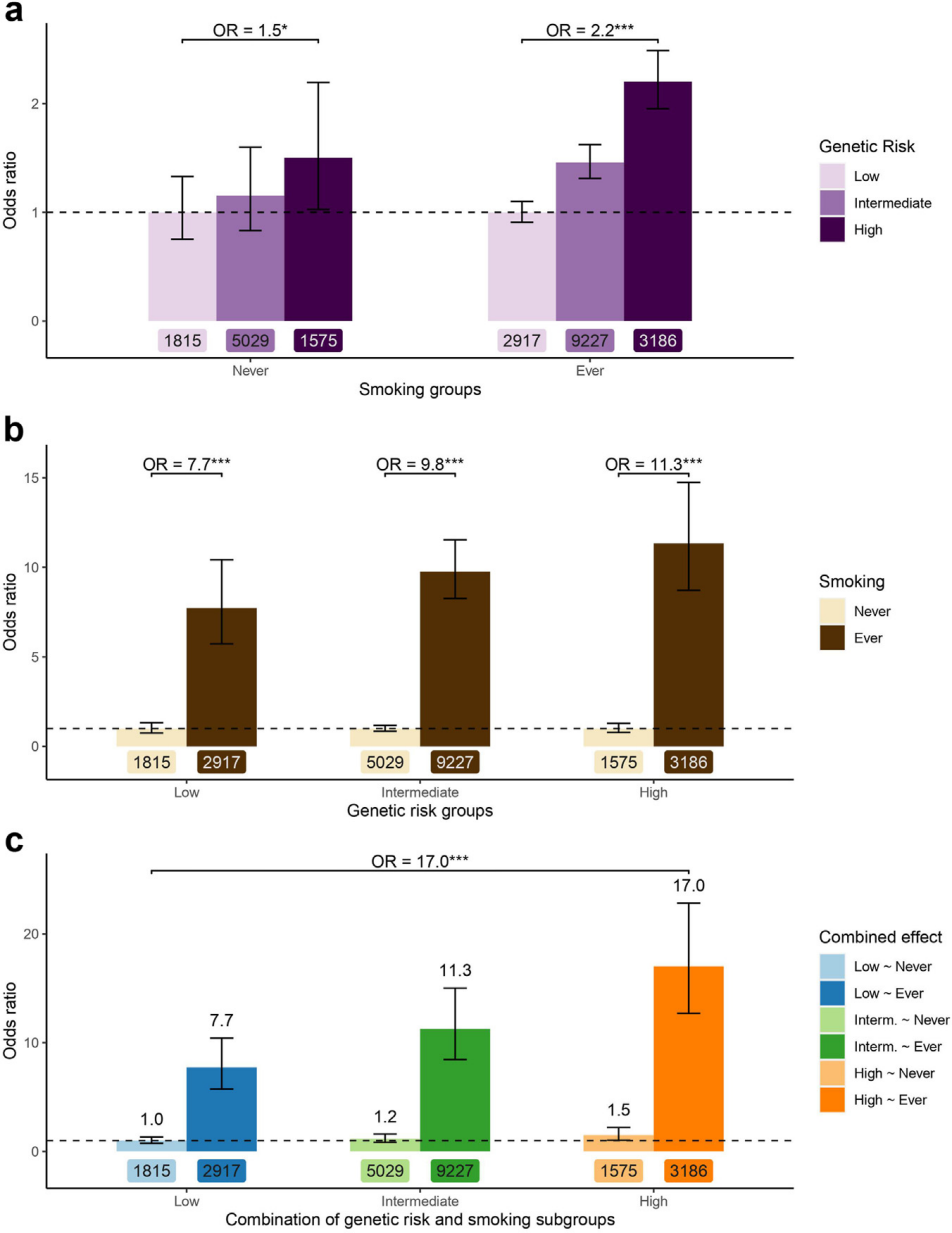
The interplay of PRS and smoking in lung cancer risk assessment. a) Odds ratios of lung cancer associated with the PRS within smoking subgroups. b) Odds ratios of lung cancer associated with smoking within genetic risk categories defined by the PRS. c) Odds ratios of lung cancer by combinations of PRS and smoking subgroups. Never smokers at low genetic risk is set as the reference (OR = 1). Number of subjects are indicated below each bar. Genetic risk groups were defined as low (<20th percentile), intermediate (20–80th percentile) and high (>80th percentile) PRS. Horizontal dashed line set at OR of 1. ∗P < 0.05, ∗∗∗P < 1E-10 [logistic regression].

The PRS results may differ by smoking status. Accordingly, the effects of PRS within smoking subgroups as well as the impact of smoking within PRS risk categories were evaluated. In individuals who never smoked, we observed only a significant increase in lung cancer risk in the high vs. low genetic risk groups (Fig. 4a). Compared to individuals with low genetic risk, OR of 1.15 (95% CI = 0.83–1.60, P = 0.389 [logistic regression]) and 1.50 (95% CI = 1.03–2.19, P = 0.036 [logistic regression]) were observed in individuals at intermediate and high genetic risk, respectively. This suggests that individuals who never smoked with a PRS in the top quintile had a 1.5-fold increased odds of lung cancer compared with those in the bottom PRS quintile. In individuals who ever smoked, a gradual and statistically significant increase in lung cancer risk was observed at intermediate genetic risk (OR = 1.46, 95% CI = 1.31–1.62, P = 4.18E-12 [logistic regression]) and high genetic risk (OR = 2.2, 95% CI = 1.95–2.48, P = 1.39E-37 [logistic regression]) compared to the low genetic risk. Accordingly, in this smoking subgroup, individuals with a PRS in the top quintile had about 2-fold increased risk of lung cancer compared with those in the bottom PRS quintile (Fig. 4a).

Lung cancer risk associated with smoking was then evaluated in individuals at low, intermediate and high genetic risk. Fig. 4b shows that smoking increased the risk of cancer in all genetic risk groups. Smoking within each PRS risk category was associated with roughly 10-fold increased risk of lung cancer, i.e. OR of 7.73 (95% CI = 5.73–10.42, P = 7.26E-41 [logistic regression]) in the low genetic risk, OR of 9.76 (95% CI = 8.62–11.53, P = 1.50E-158 [logistic regression]) in the intermediate genetic risk, and OR of 11.33 (95% CI = 8.71–14.74, P = 2.51E-73 [logistic regression]) in the high genetic risk. This suggests a relatively consistent risk associated with smoking regardless of the polygenic background. In fact, there was no statistically significant multiplicative interaction between the PRS and smoking (P_interaction_ = 0.268 [logistic regression]).

Finally, the risk of lung cancer was evaluated in subsets of individuals divided by both smoking and PRS categories (Fig. 4c). For these analyses, never smokers at low genetic risk was set as the reference. From the OR in smokers at low genetic risk mentioned above (OR = 7.73, 95% CI = 5.73–10.42, P = 7.26E-41 [logistic regression]), there was a multiplicative increased in risk in smokers at intermediate risk (OR = 11.26, 95% CI = 8.44–15.02, P = 4.51E-61 [logistic regression]) and in smokers at high genetic risk (OR = 17.02, 95% CI = 12.69–22.82, P = 6.18E-80 [logistic regression]). Together, we concluded a nearly 2-fold increased risk associated with the top compared to the bottom quintile of the PRS and a 10-fold increased risk associated with smoking, which are multiplicative resulting in a nearly 20-fold increased risk of lung cancer in smokers at high genetic risk compared to never smokers at low genetic risk. Results presented in Fig. 4 stratified by sex are provided in Supplementary Figures S8 and S9. The effects of the genetic risk groups seem consistent in males and females. The same direction of effects is also observed between sexes for smoking and the combination of smoking and PRS groups. However, the effect sizes are amplified in males and attenuated in females.

## Discussion

In this study, we put forward that PRS can sufficiently quantify genetic predisposition to lung cancer, and along with other risk factors, can improve risk stratification assessment. By generating an ethnically-matched case-control study of lung cancer, namely LORD/CARTaGENE, we provide an independent dataset from previous GWAS to benchmark previously reported PRSs and compare their performance to the lung cancer genome-wide PRS with more than 1.1 million variants. Although statistically significant, the variance explained by the various PRSs was relatively modest, and our genome-wide PRS numerically outperformed its predecessors. In addition, we demonstrated that the PRS is an independent predictor of lung cancer beyond conventional clinical risk factors with a 2-fold odds of lung cancer in individuals in the top compared to the bottom quintile of the PRS distribution. However, the diagnostic discrimination performance of the PRS was incremental over an integrated lung cancer risk model. In regard to smoking, the main risk factor of lung cancer, all PRS groups experienced an increase in risk of approximately 10-fold, confirming that no fraction of the population is genetically protected against the smoking-related risk of lung cancer. The effects of the PRS and smoking were multiplicative as individuals with a positive smoking history and high genetic risk were characterized by a nearly 20-fold increased risk of lung cancer compared to individuals who never smoked with low genetic risk.

No new lung cancer locus was found in the LORD/CARTaGENE GWAS. The French-Canadian population is believed to have a reduced genetic pool due to its historical demographic bottleneck in the 16th and 17th centuries.^32^ The possibility of finding new loci was thus conceivable. However, only two loci reached statistical significance, which are the most robust loci previously identified in lung cancer GWAS of European populations. Our results with a sample size of 4002 cases are thus not surprising and in line with previous studies.^5^ Larger sample size may be needed to identify lung cancer loci specific for the Quebec population. This also indicates that our GWAS is suitable to combine with larger European GWAS datasets on lung cancer.

We developed a genome-wide PRS including >1.1 million variants that numerically outperformed previously published lung cancer genetic risk scores. The magnitude of improvement is, however, somewhat limited. The genome-wide PRS is associated with an OR of 1.3 per standard deviation increase. It should be recalled that the sentinel variant on chr15q25 (tagged by rs1051730, MAF of 37.9% in LORD/CARTaGENE), which is the first identified and most robust GWAS locus associated with lung cancer, has a risk allele with an OR of ∼1.3. In LORD/CARTaGENE the odds of lung cancer per risk allele is also 1.3. However, converted into a standardized PRS score, the sentinel variant alone is associated with an OR of 1.2 per 1 standard deviation increase in PRS. Accordingly, in terms of effect size (not the phenotypic variance explained), the genome-wide PRS is performing only slightly better than a single SNP. When the PRS is broken down into three genetic risk groups (top quintile, middle three quintiles and bottom quintile), the effect size of the PRS is translated into a 2-fold odds of lung cancer observed in the top compared to the bottom quintile. Interestingly, this effect holds true regardless of the smoking status.

We also demonstrated that the genome-wide PRS is an independent predictor of lung cancer beyond conventional clinical risk factors, but its diagnostic discrimination performance is incremental as part of an integrated lung cancer risk model. These results are reminiscent of previous studies. A prospective cohort in Chinese populations has demonstrated that the PRS derived from GWAS-identified loci (19-variant PRS) predicted lung cancer incident cases and was an independent effective risk stratification indicator beyond age and smoking.^7^ In UK Biobank, high genetic risk (defined by the highest quintile of a 33-variant PRS) was also independently associated with an increased risk of incident lung cancer with an estimated hazard ratio of 1.16.^15^ In terms of diagnostic discrimination performance, our genome-wide PRS with an AUC of 0.580 is very similar to the best of nine PRSs tested by Lebrett et al. with an AUC of 0.588.^17^ As part of an integrated risk model, the various PRSs evaluated in that study significantly improved the discrimination performance of the model, but with AUC increases in the range of 0.002–0.015, which we interpret as incremental and similar to our study.

It is well known that smoking increases the risk of lung cancer by approximately 10-fold.^33^ In this study, we confirmed that the odds of lung cancer are roughly the same within groups defined based on their polygenic susceptibility. This indicates that no fraction of individuals is genetically protected against the smoking-related risk of lung cancer. It also confirms that smoking is much more impactful in determining the risk of lung cancer compared to genetic predisposition currently captured by the best-possible PRS. However, the genetic risk groups are still of clinical relevance. First, the PRS predicting effect is not as dominant as smoking, but within the context of our ethnically matched case-control study, it is equally meaningful compared to other lung cancer predictors such as age and sex (Table 3). Second, the joint multiplicative effect of genetic risk and smoking increases the odds of lung cancer by nearly 20-fold. This indicates that the risk of lung cancer in the presence of smoking doubles in those at high genetic risk. The extent of risk stratification benefits associated with the lung cancer-specific PRS developed in this study thus seems context-dependent. For individual-level risk prediction, the PRS seems more clinically meaningful in ever compared to never smokers.

Recent studies reported a submultiplicative interaction between smoking and PRS on lung cancer risk.^19^^,^^20^ This implies a stronger effect of the PRS in individuals who had never smoked, but also a stronger effect of smoking in the low genetic risk group. Our results are different and more in line with other studies.^15^^,^^18^ In European participants of the UK Biobank, Zhang et al.^15^ demonstrated that the hazard ratio (HR) of lung cancer was progressively increased in former and current smokers within each genetic risk group. In addition, the association between smoking and lung cancer appeared to increase with increasing genetic risk. However, no significant interaction between the PRS and smoking (defined by status or pack-years) was observed on lung cancer incidence. Note that high genetic risk was not associated with incident lung cancer among never smokers, but conferred a two-fold higher risk among current smokers. Also, in the UK Biobank study, Zhu et al.^18^ reported that the HR of lung cancer was progressively increased in light and heavy smokers, but the effects increased with increasing genetic risk. In addition, they observed a significant additive interaction between the genetic risk and smoking on incident lung cancer. Importantly, this interaction between PRS and smoking on lung cancer incidence was not observed in the China Kadoorie Biobank, suggesting ethnic difference in smoking-related risk of lung cancer. Other differences across these studies include prevalent vs. incident lung cancer cases, lung cancer overall vs. a more specific histological subtype, definition of smoking exposure, and statistical models with categorical vs. continuous variables. More studies are needed to delineate the putative interaction and direction of effect between PRS and smoking across different ethnic groups, sex and histology.

We also noted that the effect sizes of the PRS are slightly, but progressively increasing in the extreme tails of the PRS. We may thus have a smaller fraction of individuals at the end-tail of the PRS distribution (PRS >99th percentile tested in this study) with an odds ratio equivalent to *BRCA1* (OR = 10.57) and *BRCA2* (OR = 5.85) mutations in the field of breast cancer.^34^ For coronary heart disease, it was demonstrated that some individuals carry a polygenic inheritance with an effect on risk similar to mutations causing monogenic forms of the disease.^35^ Our exploratory analyses at the extreme ends of the PRS distribution suggest that this may also be the case for lung cancer, but this will require further validation with a larger sample size. In addition, more studies will be needed for establishing the upper and lower PRS limits to delineate the low and high genetic subgroups for population screening as well as to identify individuals with risk equivalent to monogenic mutations.

For the polygenic architecture of lung cancer, the most near-term clinical application of GWAS results is a PRS. The key question is: do we have a mature PRS ready for clinical implementation? Here we have specifically evaluated the predicting ability and diagnostic discrimination of the PRS in a retrospective case-control cohort. Although statistically significant and independent from other lung cancer risk factors, the effect size and discrimination performance of the PRS in delineating cases and controls do not justify its clinical utility. Further improvements are needed to obtain a mature PRS ready for clinical implementation. In the study, the SNP-based heritability was estimated at 18.5%, which represents the proportion of phenotypic variance explained by common genetic variants assayed and imputed by GWAS array. Higher explained heritability, which will be achieved with continuing progress in elucidating the GWAS polygenicity as well as uncovering rare and low-frequency variants in high/moderate-penetrance genes associated with lung cancer, will improve the predictive accuracy of PRS. It should be noted that in this study, we have tested the PRS in the context of disease diagnosis. Other clinical applications of the PRS are under investigation in the field of lung cancer. Potential downstream utilities of the PRS include the optimization of lung cancer screening in order to delineate true and false positive lung cancer cases, improve the management of indeterminate pulmonary nodules, and inform optimal timing for low-dose CT screening. Specific studies for these intended purposes will be needed.

This study has limitations. This is a retrospective case-control study and lung cancer cases were all patients who underwent curative intent resection with a majority of early-stage disease and they may not represent all patients with lung cancer. Analyses were restricted to White British and French-Canadian populations. Ethnic differences in genetic risk and its interaction with smoking were recently reported^18^ and our results may not be transposable to other populations. Controls from CARTaGENE were about 10 years younger than cases from the LORD cohort. During a 10-year period, a fraction of the 20,010 controls are likely to develop lung cancer in the future, which may result in some misclassification. However, the effect of misclassification is likely to be low knowing the incidence of lung cancer in our population, which is estimated at about 1% for a 10-year period based the Canadian Cancer Statistics (https://cancer.ca/en/research/cancer-statistics). Accordingly, the minimal loss of power that may be caused by this misclassification is compensated by the large sample size offered by the CARTaGENE cohort (in contrast to using controls of the same age, but with a sample size similar to cases, n∼4000).

In conclusion, we have generated a genome-wide PRS that outperformed previous genetic risk scores. This PRS is independently associated with lung cancer and its effect in an integrated lung cancer risk model is incremental. Smoking increases the risk of lung cancer regardless of the polygenic background, but the risk of lung cancer doubles in smokers in the top quintile compared to the ones in the bottom quintile of the PRS distribution. Further improvements in lung cancer PRS are needed to reach clinical readiness.

## Contributors

ST, PJ, and YB designed and supervised the study. CR, CL, ASL, SM, FM, CC, PD, and PJ recruited patients and performed clinical assessment. NG, VSA, DKB and CH performed the experiments. VB, HDM, ZL, SR, AE, SB, EV, PM, ST and YB performed the analyses. VB, VSA and SR prepared the Tables and Figures. MM, BJA, PJ and YB provided resources. YB wrote the first draft of the manuscript and all authors contributed and edited the final version. All authors read and approved the final version of the manuscript. VB and YB have accessed and verified the underlying data.

## Data sharing statement

The genome-wide PRS developed in this study is available in the PGS Catalog (www.pgscatalog.org, score ID: PGS004860). The statistical code needed to reproduce the results in the article is available upon request.

## Declaration of interests

B. Arsenault received research grants from Silence Therapeutics, Pfizer and Eli Lilly; received consulting fees from Silence Therapeutics, Novartis, Eli Lilly and Editas Medicine. C. Labbé received consulting fees from Amgen, AstraZeneca, Bristol-Myers-Squibb, EMD Serono, Jazz Pharmaceuticals, LEO Pharma, Lilly, Merck, Pfizer, Roche and Sanofi Genzyme. S. Martel received research grants the Ministry of Health province of Quebec and Canadian Partnership Against Cancer. Y. Bossé has received research grants from the IUCPQ Foundation. The remaining authors declare no conflict of interest.
