# Supplementary material for: Polygenic inheritance and its interplay with smoking history in predicting lung cancer diagnosis: a French-Canadian case-control cohort
Source: eBioMedicine. 2024 Jul 5;106:105234. doi: 10.1016/j.ebiom.2024.105234 (PMC11282926; doi:10.1016/j.ebiom.2024.105234)
Supplement: Supplementary Figures [file mmc1.docx]

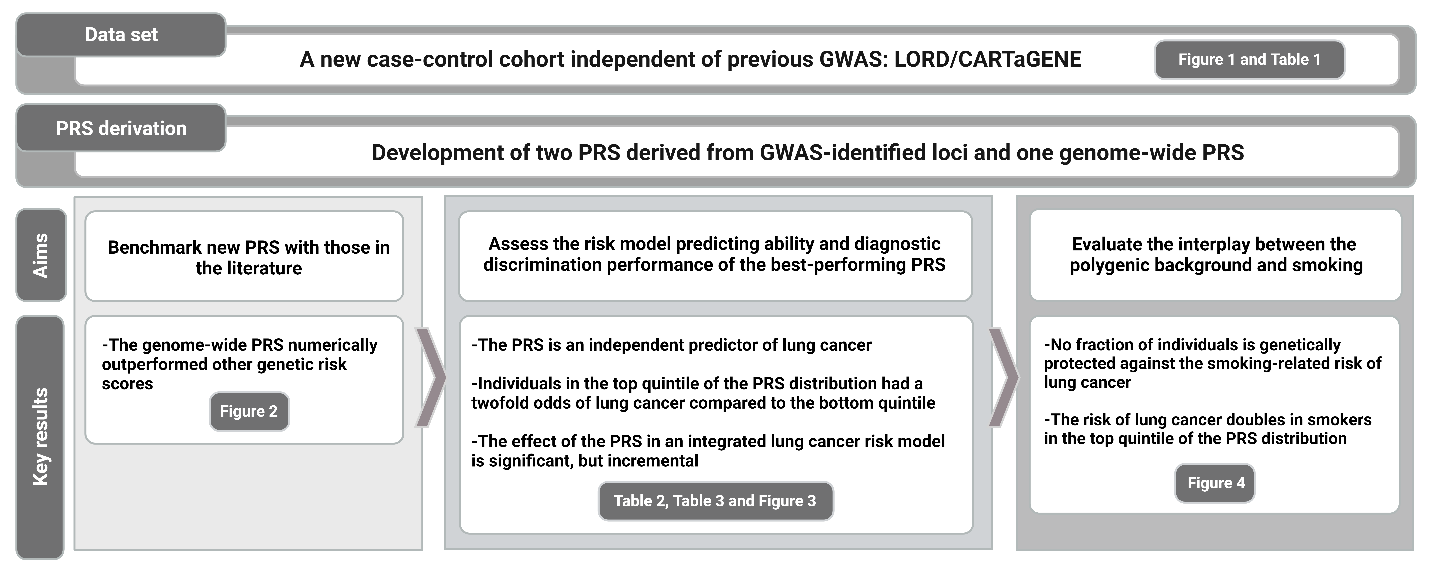


**Supplementary Figure 1**. Study workflow and main results.


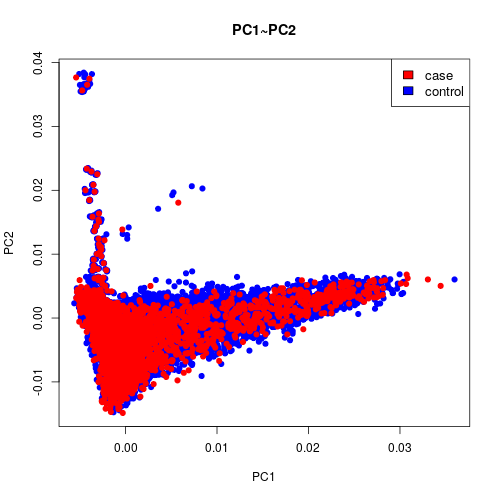


**Supplementary Figure 2**. Principal component plot to evaluate the ethnicity matching for 4,002 cases from LORD and 20,010 controls from CARTaGENE.


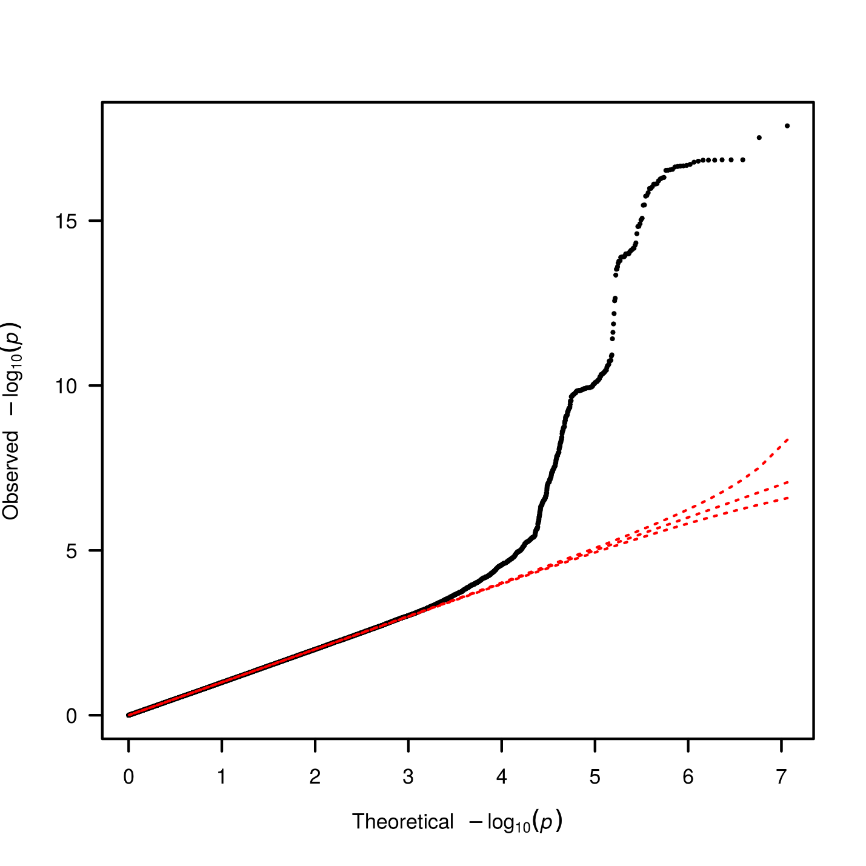


**Supplementary Figure 3**. Quantile-quantile plot of test statistics generated by the GWAS in LORD/CARTaGENE in 4,002 cases and 20,010 controls including 11,547,025 genetic variants.


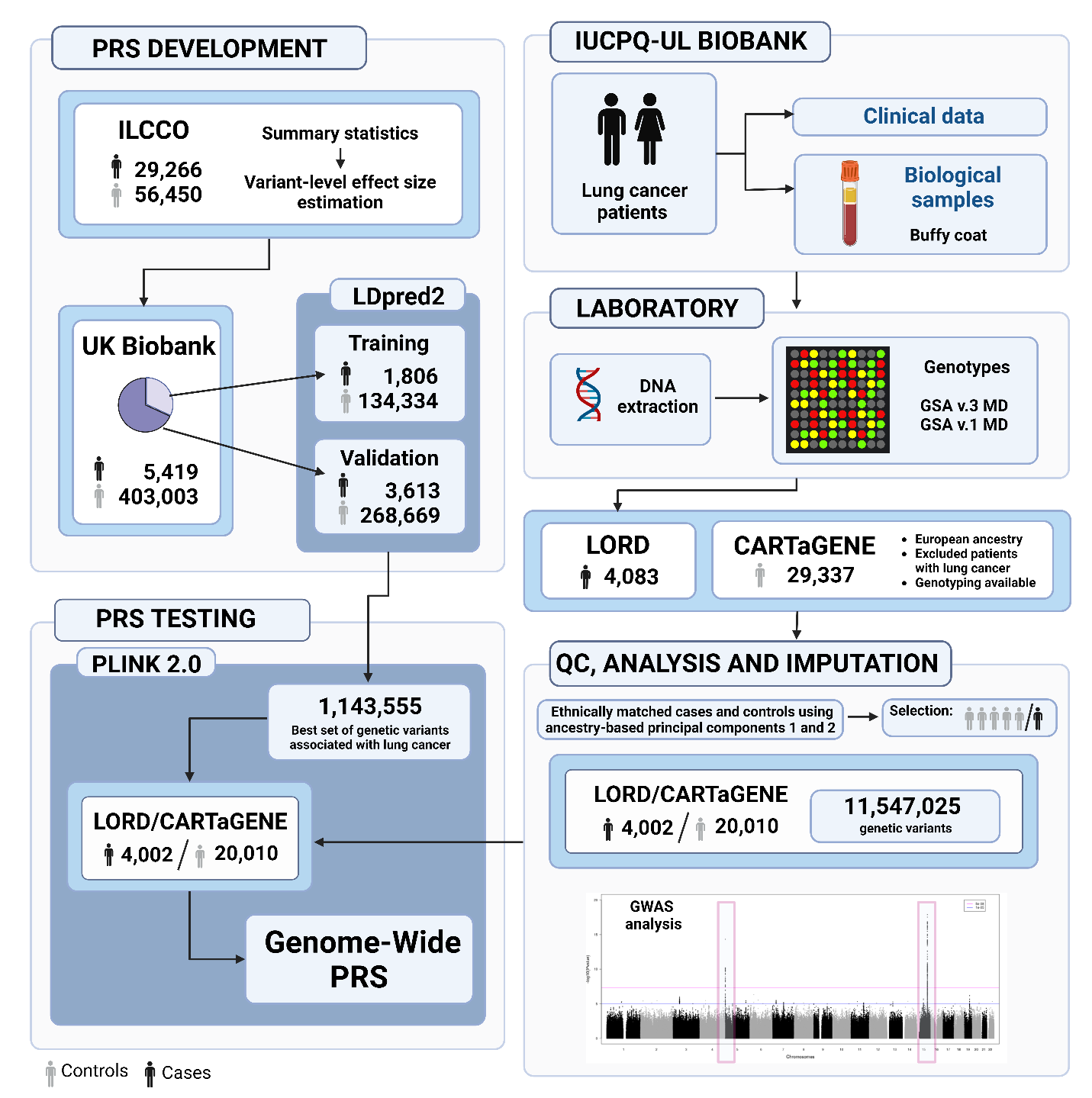


**Supplementary Figure 4**. Workflow to develop the genome-wide PRS in LORD/CARTaGENE.


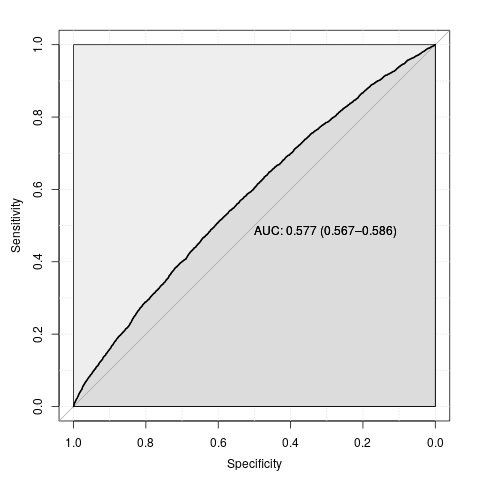


**Supplementary Figure 5**. Receiving operating characteristic curve showing the disease discrimination performance of the genome-wide PRS in the validation set (3,613 lung cancer cases and 268,669 controls) of UK Biobank.


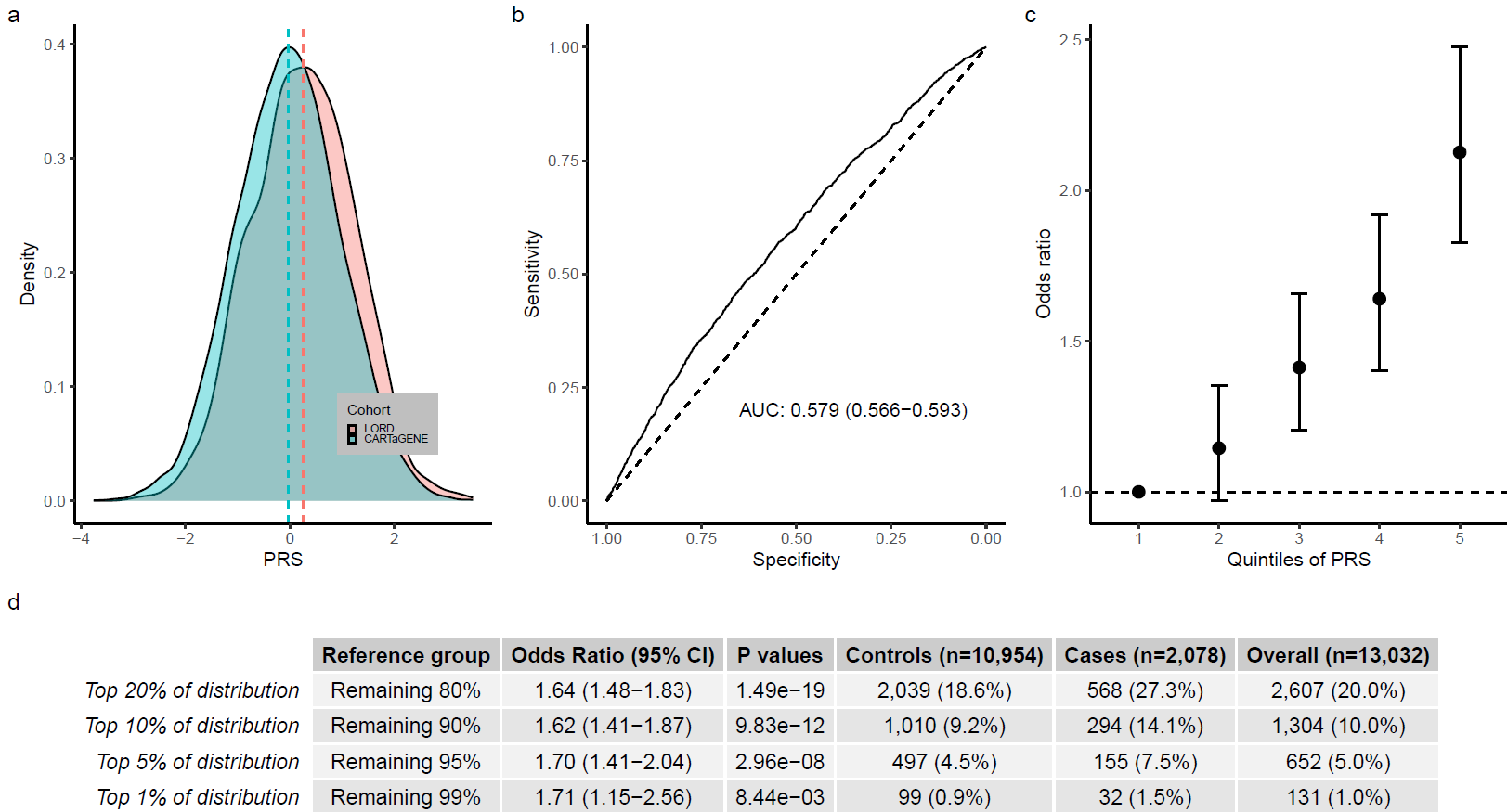


**Supplementary Figure 6**. Distribution and association of the genome-wide PRS with lung cancer in females from LORD/CARTaGENE. A) Distribution of PRS among cases and controls. B) Receiving operating characteristic curve showing the value of the PRS at discriminating between lung cancer cases and controls. C) Odds ratio of lung cancer per quintile increase in the PRS along with 95% confidence intervals. Quintile 1 as the reference including 20% of individuals with the lowest PRS. D) Risk of lung cancer according to different genome-wide PRS thresholds. All panels present univariate analysis.


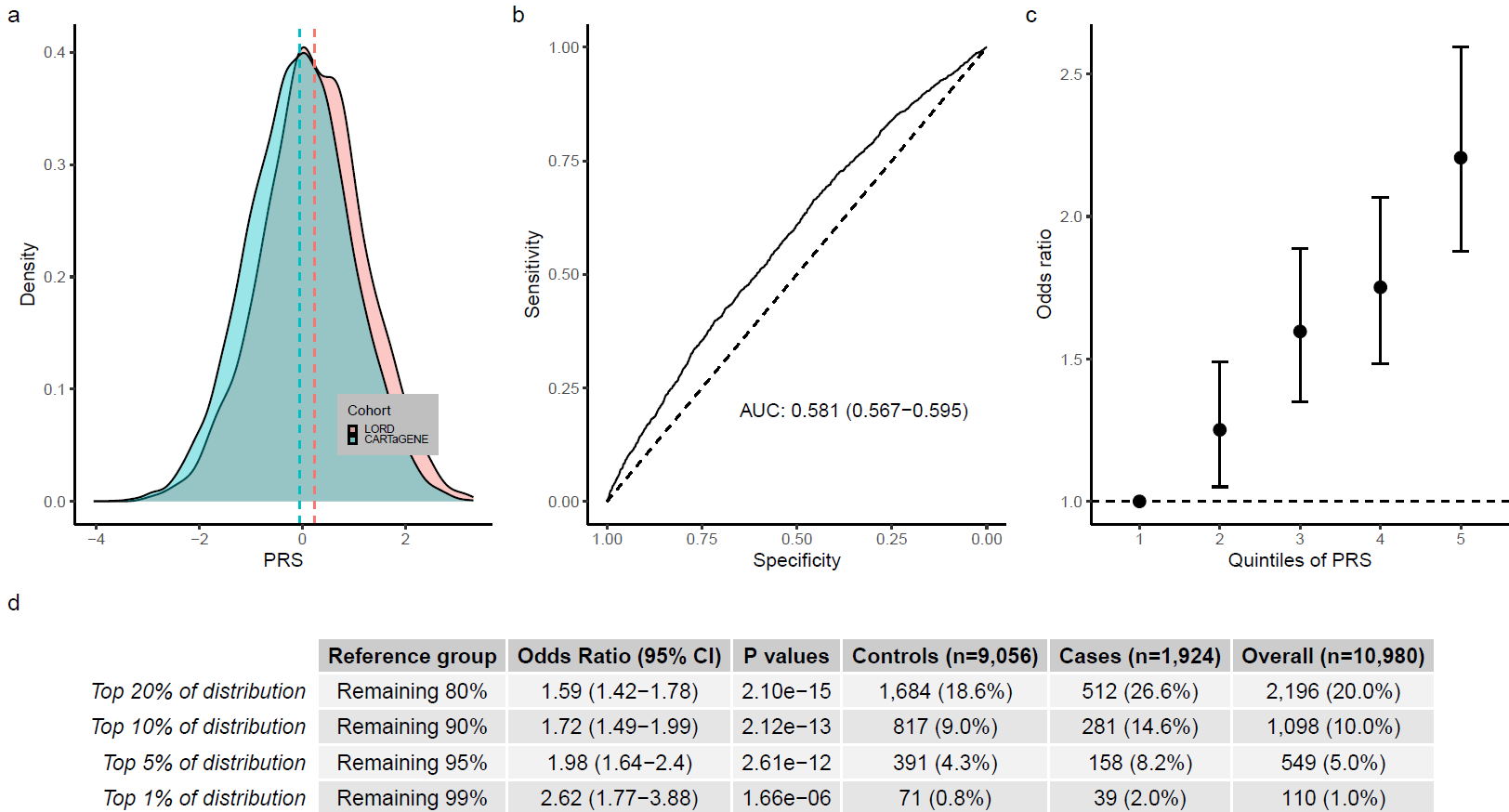


**Supplementary Figure 7**. Distribution and association of the genome-wide PRS with lung cancer in males from LORD/CARTaGENE. A) Distribution of PRS among cases and controls. B) Receiving operating characteristic curve showing the value of the PRS at discriminating between lung cancer cases and controls. C) Odds ratio of lung cancer per quintile increase in the PRS along with 95% confidence intervals. Quintile 1 as the reference including 20% of individuals with the lowest PRS. D) Risk of lung cancer according to different genome-wide PRS thresholds. All panels present univariate analysis.


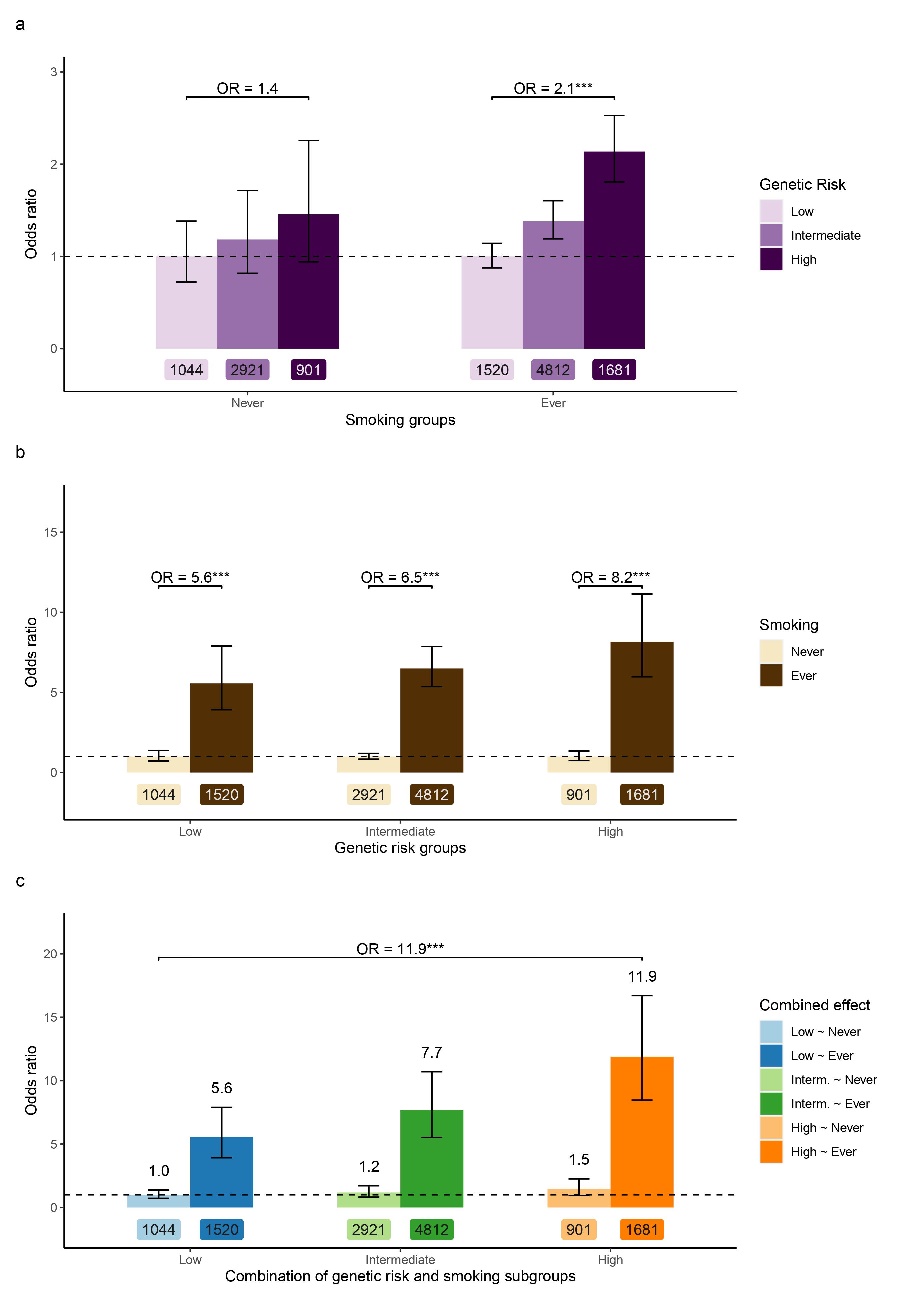


**Supplementary Figure 8**. The interplay of PRS and smoking in lung cancer risk assessment in females. A) Odds ratios of lung cancer associated with the PRS within smoking subgroups. B) Odds ratios of lung cancer associated with smoking within genetic risk categories defined by the PRS. C) Odds ratios of lung cancer by combinations of PRS and smoking subgroups. Never smokers at low genetic risk is set as the reference (OR=1). Number of subjects are indicated below each bar. Genetic risk groups were defined as low (<20^th^ percentile), intermediate (20-80^th^ percentile) and high (>80^th^ percentile) PRS. Horizontal dashed line set at OR of 1. *P < 0.05, ***P < 1E-10.

1
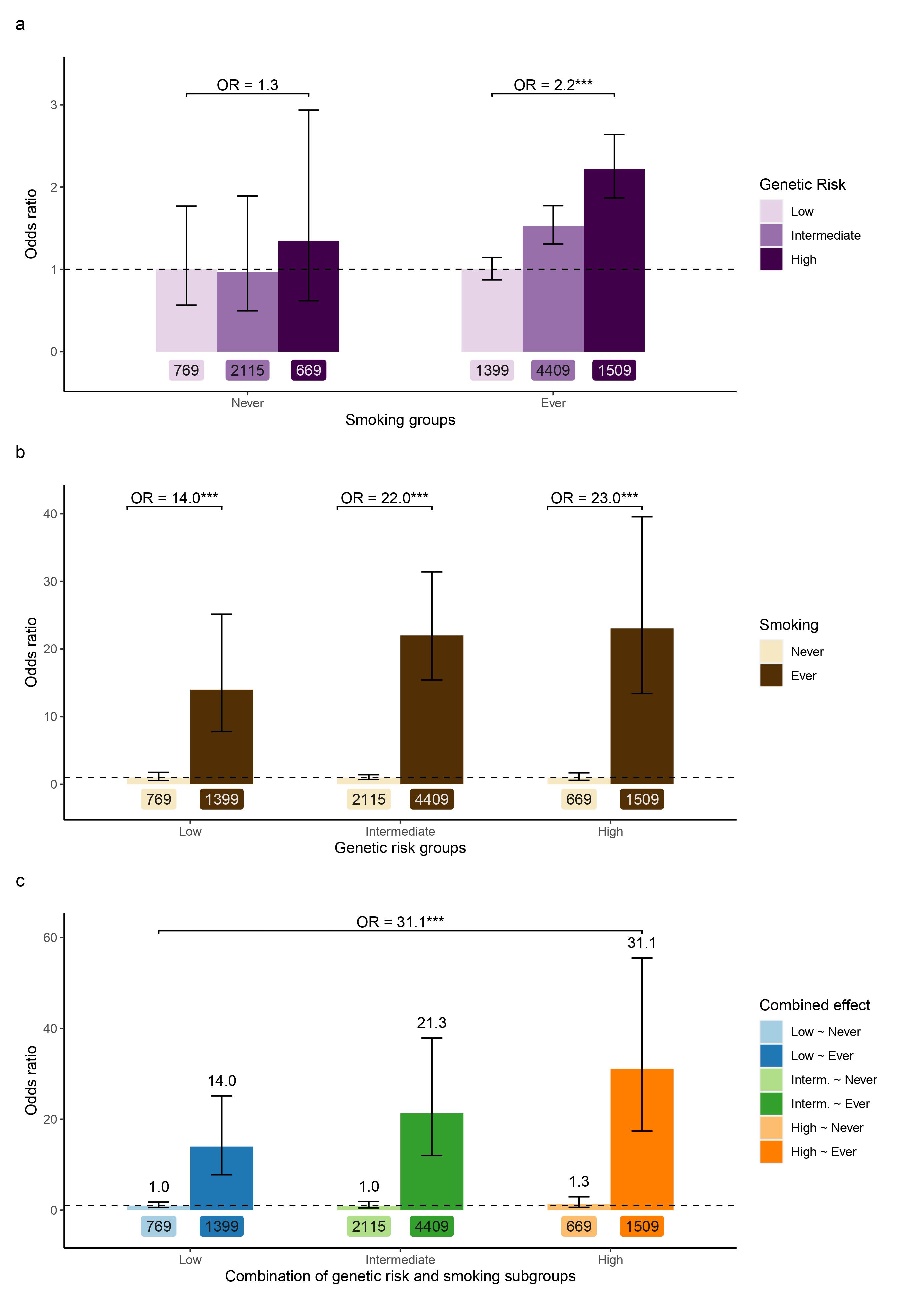


**Supplementary Figure 9**. The interplay of PRS and smoking in lung cancer risk assessment in males. A) Odds ratios of lung cancer associated with the PRS within smoking subgroups. B) Odds ratios of lung cancer associated with smoking within genetic risk categories defined by the PRS. C) Odds ratios of lung cancer by combinations of PRS and smoking subgroups. Never smokers at low genetic risk is set as the reference (OR=1). Number of subjects are indicated below each bar. Genetic risk groups were defined as low (<20^th^ percentile), intermediate (20-80^th^ percentile) and high (>80^th^ percentile) PRS. Horizontal dashed line set at OR of 1. *P < 0.05, ***P < 1E-10.
